# Optimizing the Handling of Cancellous Bone Grafts to Preserve Their Osteogenic Potential in Craniofacial Surgery

**DOI:** 10.3390/ijms26094255

**Published:** 2025-04-30

**Authors:** Cheng-Feng Chu, Chun-Yee Ho, Chia-Hsuan Tsai, Chien-Tzung Chen, Chih-Hao Chen

**Affiliations:** 1 Department of Plastic and Reconstructive Surgery, Keelung Chang Gung Memorial Hospital, Keelung 204, Taiwan; piggy310@cgmh.org.tw (C.-F.C.); chunyeeho@gmail.com (C.-Y.H.); chtsai0715@cgmh.org.tw (C.-H.T.); 2 Division of Plastic and Reconstructive Surgery, Department of Surgery, Linkou Chang Gung Memorial Hospital, Taoyuan 333, Taiwan; ctchenap@cgmh.org.tw

**Keywords:** cancellous bone grafts, bone viability, osteogenesis, handling time, storage temperature

## Abstract

Cancellous bone grafts are essential in orthopedic and plastic surgeries due to their osteoconductive and osteoinductive properties. However, handling, storage, and preservation challenges impact their viability and effectiveness in bone healing. This study assessed the effects of handling time, storage temperature, and preservation solutions on bone graft viability and osteogenesis using in vitro and in vivo models. Handling times exceeding 10 min significantly reduced cell viability, with 4 °C storage proving superior to 23 °C and 37 °C. In vivo, grafts stored at 4 °C showed enhanced bone regeneration, with PRP-treated grafts demonstrating greater osteogenic potential compared to those stored in blood or PBS. Micro-CT and histological analyses confirmed superior bone volume and tissue integration with PRP, particularly in older grafts. These findings underscore the importance of optimizing perioperative handling protocols. Storage at 4 °C and preservation in PRP emerge as promising strategies for improving bone graft outcomes in clinical applications.

## 1. Introduction

Cancellous bone grafts have revolutionized plastic reconstruction and orthopedic surgery, providing unique structural and biological attributes that enhance bone healing and regeneration. Derived from porous tissue within long bones and vertebral bodies, these grafts play a pivotal role in clinical practice. The porous architecture of cancellous bone grafts creates an optimal environment for osteoblasts and vascular ingrowth, promoting new bone formation and graft integration. Their dual role as osteoconductive and osteoinductive agents positions them as effective tools for treating bone defects from various sources. The growth factors present in cancellous bone grafts, including bone morphogenetic proteins (BMPs), transforming growth factor-beta (TGF-β), and platelet-derived growth factors (PDGFs), accelerate healing and improve clinical outcomes [1].

However, challenges arise in graft manipulation and preservation. Cellular demise within the graft due to delayed implantation is a concern. To address this, meticulous techniques and protocols are essential during graft handling, storage, and implantation to maintain cellular integrity and optimize bone healing. Despite extensive research, a conclusive consensus on optimal handling remains elusive [2,3,4,5,6,7,8,9,10,11,12,13,14,15,16,17,18,19,20,21,22]. Shorter handling intervals are generally recommended, and storage at various temperature settings has been explored. Further research and standardization are needed to establish guidelines for bone graft handling and storage.

The emerging evidence suggests that handling cancellous bone grafts beyond 10 min significantly reduces cell viability and osteogenic potential. Studies show that apoptotic cells triple within the first 5 min, with Runx2 and Osterix expression declining after 10–30 min, leading to impaired bone regeneration [23]. Additionally, in vitro assessments reveal a sharp decline in viable osteoblast-like cells beyond 10 min, reinforcing the need for rapid implantation or protective storage [23]. These findings highlight the importance of minimizing handling time to maintain graft viability and optimize bone healing.

Additionally, innovative approaches to bone grafting continue to emerge. For instance, ASC-iPSCs have shown superior osteogenic differentiation potential, representing a promising advancement in bone tissue engineering [24]. Similarly, techniques such as 3D-printed scaffolds seeded with BMSCs offer a scaffold-based approach that facilitates bone regeneration in challenging anatomical defects [25]. Despite these advances, the unique advantages of autogenous grafts remain unmatched, as they combine osteogenesis, osteoinduction, and osteoconduction in a single procedure [1]. Although bioinert materials such as stainless steel and alumina are commonly used in surgical applications due to their reproducibility, their inability to fuse effectively with host bone limits their long-term success [26]. Advances in tissue engineering have sought to address this through the incorporation of osteogenic cells and bioactive proteins, creating materials with enhanced osteoinductive properties [27].

Despite the well-established importance of cell viability in graft success, the impact of brief intraoperative handling times (e.g., 5–30 min) at different temperatures remains poorly characterized. Most of the existing studies focus on cryopreservation techniques or extended storage durations rather than the critical short-term exposure that occurs during surgery. Additionally, while preservation solutions such as saline, blood, and platelet-rich plasma (PRP) have been used in clinical practice, comparative analyses on their efficacy in maintaining graft viability have been sparse.

This study aims to address these gaps by systematically evaluating the effects of handling time, temperature, and preservation medium on the viability and osteogenic potential of cancellous bone grafts. By integrating in vitro and in vivo models, we seek to establish evidence-based guidelines for optimizing bone graft handling protocols to enhance clinical outcomes in craniofacial and orthopedic surgery.

## 2. Results

### 2.1. Handling Time

**Figure 1:**
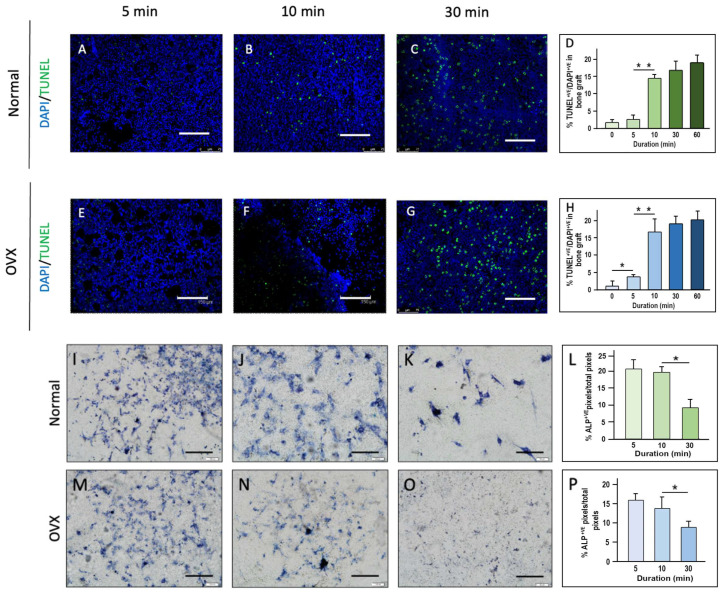
Cell viability, apoptosis, and ALP activity in normal and osteoporotic bone grafts at 23 °C. Live/dead staining was performed on normal (**A**–**C**) and osteoporotic (**E**–**G**) bone grafts at 5, 10, and 30 min. Scale bar = 150 μm. Apoptotic cell percentages at 0, 5, 10, 30, and 60 min are shown in bar charts (**D**,**H**), based on TUNEL and DAPI staining. Representative ALP staining images of normal (**I**–**K**) and osteoporotic (**M**–**O**) bone grafts at 0, 10, and 30 min. Scale bar = 200 μm. Bar charts (**L**,**P**) show ALP-positive pixel quantification at these time points. * *p* < 0.05–0.01 ** *p* < 0.01–0.001.

Live and dead cell staining revealed a general increase in apoptotic cells with longer handling times in both normal and osteoporotic bone grafts at 23 °C. Osteoporotic bone grafts consistently exhibited more apoptotic cells compared to normal grafts across all handling times. The increase in apoptotic cell counts became significantly pronounced when handling time exceeded 10 min, indicating a critical threshold for maintaining cellular viability (Figure 1).

Alkaline phosphatase (ALP) staining demonstrated higher ALP activity in normal bone grafts compared to osteoporotic grafts across all handling durations. Prolonged handling times correlated with a reduction in ALP activity, with a notable and statistically significant decrease observed beyond 10 min. These findings suggest that extended handling adversely affects the osteogenic potential of the grafts (Figure 1). Additionally, to visualize cellular viability changes during graft handling, we performed live/dead staining on human bone grafts from two donors (a 21-year-old male and a 65-year-old male), as presented in Supplementary Video S1. The video highlights the progressive decline in cell viability over a 30-min handling period.

**Figure 2:**
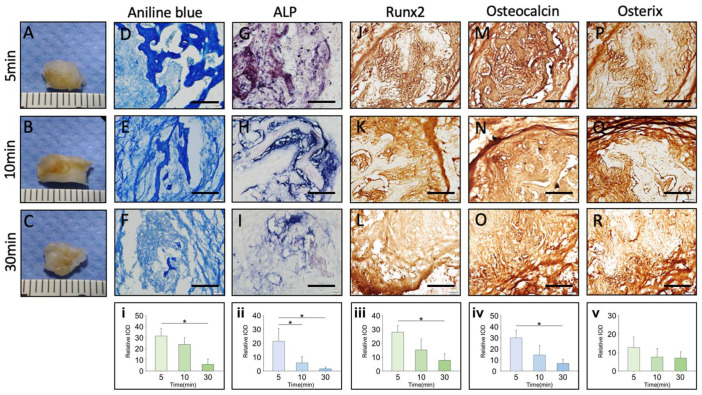
Immunohistochemical staining of subcutaneous implantation of bone graft in nude mice at postoperative day 21 (PID21). Bone grafts handled at 23 °C for different durations were assessed. Gross Images: Bone grafts after graft handling for 5, 10, and 30 min (**A**–**C**). Scale bar = 1 mm. Aniline blue staining for grafts handled for 5, 10, and 30 min (**D**–**F**). ALP Staining for grafts handled for 5, 10, and 30 min (**G**–**I**). RUNX2 Staining for grafts handled for 5, 10, and 30 min (**J**–**L**). Osteocalcin staining for grafts handled for 5, 10, and 30 min (**M**–**O**). Osterix staining for grafts handled for 5, 10, and 30 min (**P**–**R**). Scale bar = 300 μm. Bar charts (**i**–**v**) quantify staining intensity of RUNX2, Osteocalcin, and Osterix in grafts handled for 5, 10, and 30 min. * *p* < 0.05–0.01.

Immunohistochemical staining of bone graft at post-implantation day 21 (PID 21) demonstrated superior collagen deposition, increased osteoblastic activity, and enhanced osteogenic transcriptional activity in grafts handled for shorter durations at 23 °C. Specifically, staining for Runx2, Osteocalcin, and Osterix revealed more intense and widespread expression in grafts handled for 5 and 10 min, while those handled for 30 min exhibited noticeably weaker staining. These findings suggest that shorter handling durations better preserve the osteogenic potential of cancellous bone grafts, reinforcing the importance of minimizing intraoperative exposure time to enhance bone regeneration (Figure 2).

Quantitative analysis (Figure 2i–v) further confirmed these trends, showing significantly higher osteogenic marker expression in shorter handling durations, supporting the importance of minimizing intraoperative handling times to preserve osteogenic viability.

### 2.2. Handling Temperature

**Figure 3:**
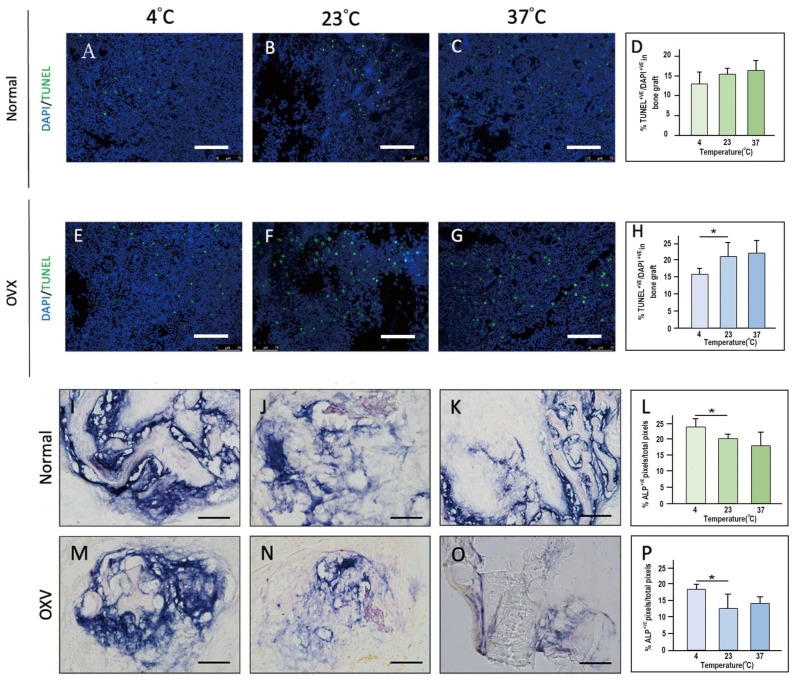
Cell Viability, Apoptosis, and ALP Activity in Normal and Osteoporotic Bone Grafts at Different Temperatures. Live/Dead Cell Staining: Normal (**A**–**C**) and osteoporotic (**E**–**G**) bone grafts handled for 10 min at 4 °C, 23 °C, and 37 °C. Scale bar = 150 μm. Apoptotic cell percentages are shown in bar charts (**D**,**H**) based on TUNEL and DAPI staining. Alkaline Phosphatase (ALP) Staining at PID21: Representative ALP staining of normal (**I**–**K**) and osteoporotic (**M**–**O**) bone grafts stored at 4 °C, 23 °C, and 37 °C. Scale bar = 300 μm. Bar charts (**L**,**P**) quantify ALP-positive pixels relative to total pixels. * *p* < 0.05–0.01.

When bone grafts were handled for 10 min at different temperatures, live/dead cell staining revealed an increase in apoptotic cells as temperature increased. Osteoporotic grafts exhibited consistently higher apoptosis rates than normal grafts at all temperatures. The lowest apoptosis levels were observed at 4 °C, while significantly higher apoptosis was noted at 23 °C and 37 °C, indicating a protective effect of lower temperatures on cellular viability (Figure 3).

At post-implantation day 21 (PID 21), ALP staining demonstrated greater osteoblastic activity in grafts stored at 4 °C, followed by 23 °C, with the lowest activity observed at 37 °C. Notably, the 23 °C group showed moderate ALP expression, suggesting partial preservation of osteogenic potential but significantly less than the 4 °C group. These results emphasize the importance of temperature control in maintaining the osteogenic viability of cancellous bone grafts (Figure 3).

### 2.3. Preservation Medium

Immunohistochemical (IHC) staining of subcutaneous implantation of osteoporotic bone graft in nude mice demonstrated distinct differences in osteogenic activity across different preservation media (PBS, blood, PRP) and handling temperatures (4 °C vs. 23 °C) after 10 min.

**Figure 4:**
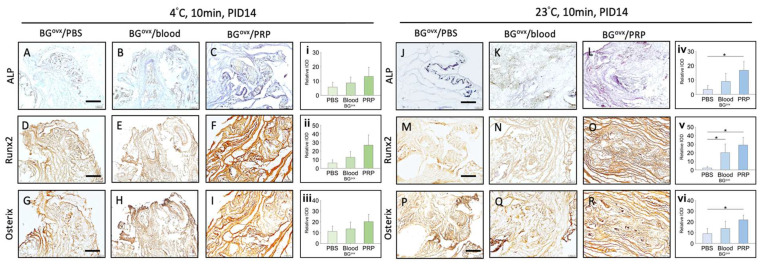
IHC Staining of subcutaneous implantation of osteoporotic bone graft in nude mice at 4 °C and 23 °C after 10 min. ALP Staining: Osteoporotic bone grafts handled in PBS, blood, and PRP at 4 °C (**A**–**C**) and 23 °C (**J**–**L**). Runx2 Staining: Osteoporotic bone grafts handled in PBS, blood, and PRP at 4 °C (**D**–**F**) and 23 °C (**M**–**O**). Osterix Staining: Osteoporotic bone grafts handled in PBS, blood, and PRP at 4 °C (**G**–**I**) and 23 °C (**P**–**R**). Scale bar = 200 μm. Bar charts (**i**–**vi**) quantify staining intensity of ALP, Runx2, and Osterix expression in bone grafts preserved in PBS, blood, and PRP at PID 14. * *p* < 0.05–0.01.

ALP staining showed greater intensity and a more widespread distribution at 4 °C across all media, suggesting enhanced early osteoblast differentiation compared to 23 °C (Figure 4A–C,J–L).

Runx2 and Osterix staining exhibited stronger nuclear localization at 4 °C, particularly in PRP-preserved grafts, indicating increased osteogenic transcriptional activity (Figure 4D–I,M–R).

While lower temperatures (4 °C) contributed to improved osteogenic marker expression, the choice of preservation medium also played a significant role. PRP-treated samples at 4 °C displayed the most pronounced osteogenic activity, outperforming those stored in blood or PBS. In contrast, samples handled at 23 °C showed weaker and more diffuse staining, particularly in the PBS group, suggesting reduced osteoblastic activity and differentiation potential.

Quantitative analysis (Figure 4i–vi) confirms significantly higher osteogenic marker expression in PRP-preserved grafts, emphasizing that PRP preservation at 4 °C provides optimal conditions for bone graft viability.

**Figure 5:**
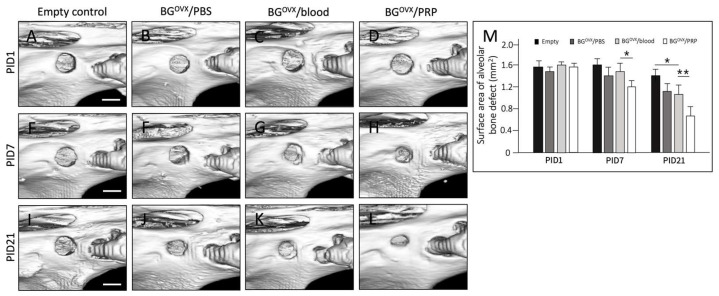
Micro-CT Analysis of Osteoporotic Rat Alveolar Defects at Different Post-Implantation Days. Representative micro-CT images of osteoporotic rat alveolar defects at post-implantation day (PID 1 (**A**–**D**), PID 7 (**E**–**H**), and PID 21 (**I**–**L**)), following bone grafting with different treatments: no solution (empty control), PBS, blood, and PRP (all handled for 10 min at 23 °C). Scale bar = 2 mm. Bar chart (**M**) showing alveolar defect surface area (mm^2^) over time (PID 1, 7, and 21) for each treatment condition. * *p* < 0.05–0.01 ** *p* < 0.01–0.001.

To further validate these findings, quantitative comparisons at post-implantation day 7 (PID 7) and PID 21 were conducted. Micro-CT analysis revealed greater bone regeneration in PRP-preserved grafts at 4 °C compared to 23 °C, correlating with higher ALP, Runx2, and Osterix expression. These results emphasize that both temperature and preservation medium contribute synergistically to graft osteogenic potential (Figure 5).

Micro-CT analysis of rat alveolar defects post-grafting demonstrated that bone grafts preserved in platelet-rich plasma (PRP) exhibited superior bone healing outcomes compared to those stored in blood, phosphate-buffered saline (PBS), or without a preservation solution. At post-implantation day 21 (PID 21), the bone volume fraction (BV/TV%) was highest in PRP-preserved grafts (48.7% ± 4.2), followed by blood-preserved grafts (39.8% ± 3.5), PBS-preserved grafts (33.4% ± 3.1), and the no-preservation group (21.6% ± 2.8) (Figure 5M).

**Figure 6:**
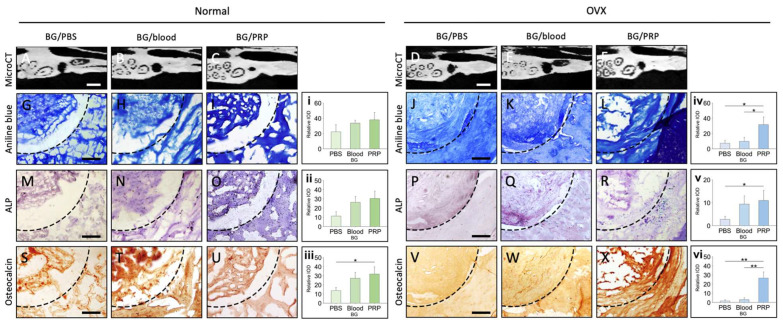
Comparison of Normal vs. Osteoporotic Bone Grafts in Normal and Osteoporotic Rat Alveolar Defects at Post-Implantation Day 21 (PID 21) with Different Solutions. Representative micro-CT images (**A**–**F**) of normal and osteoporotic rat alveolar defects at PID 21, with bone grafts handled in PBS, blood, or PRP for 10 min at 23 °C. Scale bar = 2 mm. Representative aniline blue staining (**G**–**L**) illustrating collagen deposition, ALP staining (**M**–**R**) indicating osteoblastic activity, and osteocalcin staining (**S**–**X**) showing late-stage bone formation in both normal and osteoporotic rats. Scale bar = 200 μm. Quantitative bar chart analysis of immunohistochemical staining comparing normal (**i**–**iii**) and osteoporotic (**iv**–**vi**) bone grafts preserved in PBS, blood, and PRP at PID 21. * *p* < 0.05–0.01 ** *p* < 0.01–0.001.

Micro-CT images show that PRP-preserved grafts exhibited the greatest bone volume and defect closure, indicating superior bone healing compared to blood and PBS. In contrast, PBS-preserved grafts demonstrated the least bone formation, suggesting inferior osteogenic support (Figure 6A–F).

Aniline blue staining (Figure 6G–L) highlights collagen deposition, with PRP-treated grafts displaying denser, more organized collagen fibers, particularly in normal bone grafts. In osteoporotic grafts, collagen formation appeared less robust, though PRP still provided a notable improvement over PBS.

ALP staining (Figure 6M–R), an indicator of osteoblastic activity, was most intense in PRP-treated grafts, confirming enhanced osteogenesis. Blood-preserved grafts showed moderate ALP expression, whereas PBS-treated grafts exhibited the weakest staining, indicating reduced osteogenic differentiation.

Osteocalcin staining (Figure 6S–X), a marker of late-stage bone formation, further supports these findings. PRP-treated grafts displayed strong osteocalcin expression, particularly in normal grafts, reflecting greater bone maturation and mineralization. In contrast, PBS-treated grafts showed weak and dispersed osteocalcin staining, reinforcing their limited osteogenic potential.

Quantitative analysis (Figure 6i–vi) of aniline blue, ALP, and Osteocalcin staining demonstrated that PRP-treated grafts had the highest collagen deposition, osteoblastic activity, and late-stage bone formation, reinforcing PRP’s role in maintaining bone graft viability and enhancing osteogenic potential.

## 3. Discussion

Our study evaluated the effects of handling time, storage temperature, and preservation solutions on the viability and osteogenic potential of cancellous bone grafts through in vitro and in vivo models. The findings indicate that handling time exceeding 10 min significantly increases cellular apoptosis, emphasizing the importance of completing the grafting process swiftly, ideally within this time frame. Cooler storage temperatures (4 °C) were found to preserve cell viability more effectively, particularly in OVX bone grafts, suggesting that immediate post-harvest immersion in an ice water bath could further enhance graft preservation. Additionally, young bone grafts showed superior osteogenic potential compared to OVX grafts. Furthermore, grafts stored in platelet-rich plasma (PRP) exhibited enhanced osteogenic activity compared to those stored in blood or PBS, underscoring the potential of immediately immersing grafts in PRP, or at least PBS, to promote bone regeneration, especially in OVX grafts.

Minimizing handling time is essential for maintaining cancellous bone graft viability, as prolonged exposure, even under optimal conditions, leads to a significant decline in cell survival and osteogenic potential. Studies have shown that dry storage rapidly reduces viable osteogenic cells, with cell viability dropping by ~37% after 1 h and ~63% after 4 h of dry exposure, ultimately impairing graft function and fusion success [21]. Similarly, Rocha et al. found that even 30 min of air exposure resulted in significantly more empty osteocyte lacunae, indicating extensive cell death [6]. Storage temperature also plays a role, with body temperature (37 °C) accelerating cell loss, while room temperature (~22 °C) and refrigerated conditions (~4 °C) better preserve osteoblast viability in the short term [19]. Despite these factors, time remains the most critical determinant, as Kantor et al. noted that while the grafts maintained viability for up to 2 h in controlled conditions, longer durations increase the risk of cell deterioration [22]. To optimize graft survival and bone healing outcomes, the current evidence strongly supports immediate implantation or maintaining grafts in hydrated conditions, such as saline or blood, for the shortest time possible [19,21]. Similarly, Sohn et al. found that storage of in-vitro-expanded MSCs in saline or dextrose solution for more than 2 h at 4 °C or room temperature significantly decreased viability, proliferation capacity, and differentiation potential [2].

Standard tissue bank guidelines recommend storing grafts at 1–10 °C [3]. Refrigeration is widely practiced due to concerns about microbial growth at higher temperatures [4]. Studies generally indicate better viability at lower temperatures, consistent with our results. Antonenas et al. showed that refrigerated storage (2–8 °C) significantly reduced the loss of viable CD34+ cells in fresh bone marrow and peripheral blood stem cells compared to room temperature (18–24 °C) at 24, 48, and 72 h [5]. Hamidreza et al. reported that histomorphometric analysis identified incubator temperatures as least favorable for goat iliac crest graft storage [6]. Hypothermic conditions also benefit bone marrow-derived mesenchymal stem cells. Ginis et al. found nearly complete cell recovery after 2 days and 85% after 4 days of hypothermic storage, indicating superior osteogenic potential at low temperatures [7].

However, some studies challenge low-temperature storage for specific tissues. Pallante et al. noted a 28% reduction in chondrocyte viability in osteochondral allografts stored at 4 °C for 28 days, while storage at 37 °C improved viability and maintained glycosaminoglycan metabolism and extracellular matrix integrity [8]. Stoker et al. demonstrated that storing osteochondral allografts at 25 °C using a proprietary technique resulted in better preservation of graft quality without increasing the risk of microbial contamination compared to storage at 4 °C with the same method [9]. Additionally, some researchers have suggested that the metabolic responses of osteochondral allografts during the transition from storage temperature to body temperature could be detrimental. Stoker et al. later found that storage at 25 °C led to significantly lower levels of inflammatory mediators (PGE2) and degradative enzymes (MMP-1, MMP-2, MMP-13), while also maintaining significantly higher viable chondrocyte density, which is critical for successful transplantation, compared to grafts stored at lower temperatures [10]. Hofmann et al. suggested that thermodisinfection at 80 °C for fresh-frozen bone resulted in cell viability comparable to traditional freezing methods [11].

The choice of storage medium significantly affects graft viability, with most studies demonstrating that moist environments are superior to dry ones. Maus et al. found significantly reduced cell counts in human cancellous bone stored dry for 2 h compared to saline or culture media [12]. Among available solutions, lactated Ringer’s (LR) is commonly used for short-term preservation [13] as it is isotonic and contains lactate, CaCl_2_, KCl, and NaCl. However, LR lacks nutrients essential for sustaining cell metabolism [14]. The research indicates that storage media enriched with nutrients are more effective at preserving cells compared to using lactated Ringer’s solution alone [15]. Nutrient-enriched solutions, such as Dulbecco’s Modified Eagle Medium (DMEM), offer improved cell preservation due to their enriched composition of vitamins, amino acids, and other metabolic supplements [28]. Teng et al. demonstrated that after two weeks, DMEM preserved 54.8% cell viability compared to 20.4% for LR [13].

Adding serum further enhances outcomes. Pennock et al. showed that fetal bovine serum significantly outperformed serum-free media in maintaining viable chondrocyte density (82.1% vs. 27.3%) and metabolic activity, as measured by proteoglycan production and cartilage density [16]. However, concerns about the ethical sourcing of fetal bovine serum [17] and potential disease transmission [18] have shifted attention toward autologous plasma or serum. Previous studies suggest that platelet-poor plasma (PPP) performs comparably or better than saline. Rocha et al. reported superior histomorphometric outcomes for rabbit bone grafts stored in PPP compared to dry conditions after 30 min [19]. Similarly, Deichichi et al. demonstrated that PPP was significantly better than saline in preserving osteocytes in rabbit calvarial bone grafts after 30 min [20]. Our findings align with these results, showing that PRP outperforms fresh blood and PBS as a preservation medium. Interestingly, Hamidreza et al. found that while dry storage was superior to LR for goat iliac crest grafts, blood emerged as the most effective medium, corroborating our conclusion that PRP offers the best preservation outcomes [6].

Our findings demonstrated that PRP effectively preserved graft viability and enhanced osteogenic potential compared to blood and PBS. These results are consistent with previous studies that have investigated other growth-factor-enriched preservation solutions. When comparing platelet-rich plasma (PRP) and recombinant growth factors (e.g., BMP-2, PDGF-BB, and FGF-2) for bone graft preservation, key differences emerge in terms of efficacy, mechanism, and clinical potential. PRP, derived from autologous blood, provides a broad spectrum of growth factors, including platelet-derived growth factor (PDGF), transforming growth factor-beta (TGF-β), and vascular endothelial growth factor (VEGF), which contribute to early-stage healing by enhancing angiogenesis and osteoblast recruitment [29,30]. However, the release of PRP’s growth factor depends heavily on preparation techniques and patient-specific variability. In contrast, recombinant growth factors, such as bone morphogenetic protein-2 (BMP-2) and fibroblast growth factor-2 (FGF-2), offer a more targeted and sustained osteoinductive effect, directly stimulating mesenchymal stem cell differentiation into osteoblasts and promoting bone matrix deposition [31,32]. Notably, BMP-2 has demonstrated superior bone formation in osteoporotic conditions where PRP’s regenerative potential is limited [31]. While FGF-2-loaded collagen membranes have shown enhanced bone density and defect closure compared to PRP, PDGF-BB has displayed limited efficacy in compromised bone healing scenarios [31,32]. Overall, while PRP supports early healing and vascularization, recombinant growth factors like BMP-2 and FGF-2 provide a stronger, more reliable osteogenic stimulus, making them preferable for severe defects or osteoporotic bone regeneration, whereas PRP remains a cost-effective and autologous alternative for minor bone graft augmentation [30].

The use of platelet-rich plasma (PRP) in bone graft preservation presents several challenges, particularly in terms of variability in preparation methods and the risk of immune reactions, which limit its consistent clinical application. Studies have demonstrated that differences in centrifugation protocols, platelet concentrations, and the presence or absence of leukocytes significantly affect PRP’s efficacy in promoting bone regeneration [33,34]. The lack of standardized preparation techniques results in inconsistent clinical outcomes, making it difficult to compare studies and establish clear guidelines for use [35]. Moreover, while PRP is considered autologous and generally biocompatible, leukocyte-rich PRP has been associated with increased inflammatory responses, and non-autologous activation agents can provoke immune-mediated adverse effects [34]. These findings highlight the need for greater protocol standardization and further clinical trials to optimize PRP formulations, minimize immune-related risks, and enhance its reliability for bone graft preservation.

Bone grafting plays an essential role in clinical practices such as bone void management, fracture treatment, and post-resection reconstruction of bone malignancies [36]. Autologous bone grafts promote skeletal regeneration via growth factors, while allografts and synthetic grafts provide structural frameworks to activate endogenous bone regeneration [37]. The optimal choice of grafting method depends on factors like patient profile, anatomical defects, and therapeutic goals, with the aim of improving bone structure and function, thereby enhancing patient quality of life. Despite the critical importance of bone grafting, there is limited understanding of the impact that intraoperative storage methods have on the grafts’ viability and function. Immediate implantation of freshly harvested grafts typically yields the best outcomes, but the role of storage conditions requires further exploration [12].

Autogenous bone grafting remains the gold standard due to its combination of mesenchymal stem cells (MSCs), which enhance osteogenesis, and growth factors that support osteoinduction. Autografts also provide a calcified osteoconductive framework, making them highly effective for treating bone defects [1]. However, challenges in donor site morbidity and availability have spurred interest in alternative strategies, such as allografts and synthetic materials. Unprocessed allografts, while effective, often require freezing or freeze-drying due to their limited shelf life. Rehydration before grafting partially restores their initial physical properties, though further innovations are needed to improve their osteogenic efficacy [38].

Despite bone grafting being the gold standard of bone regeneration, recent advancements in biomaterials and tissue engineering have introduced innovative approaches to enhancing bone regeneration. Chu et al. demonstrated the efficacy of allogeneic bone-marrow mesenchymal stem cells (BMSCs) enriched gelatin-nanohydroxyapatite cryogel scaffolds in craniofacial bone regeneration, highlighting their potential for personalized medicine applications [39]. Similarly, Glenske et al. reviewed the role of metal ions in bone tissue regeneration, emphasizing how their integration into bone substitute materials can modulate inflammatory responses and promote osteogenesis [40]. Jung et al. explored the biocompatibility of hydrofluoric-acid-passivated magnesium screws, finding improved outcomes for guided bone regeneration [41]. Flaig et al. investigated jellyfish collagen scaffolds, revealing their ability to induce anti-inflammatory macrophage responses and significant bone formation in vivo, positioning them as a viable alternative to mammalian collagen-based biomaterials [42]. Hasan et al. introduced a bioglass-based antibiotic-releasing bone void filling putty that not only eradicates bacteria but also supports bone regeneration, offering a dual benefit in treating osteomyelitis [43]. These emerging techniques provides alternatives for bone regeneration when bone grafting is unavailable.

Storage conditions, including temperature, humidity, and exposure time, significantly influence the osteogenic potential and cellular integrity of bone grafts [6]. In our study, we observed a rapid decline in cellular viability within just 10 min of storage. This finding aligns with previous reports showing that fat grafts, for instance, experience a 50% survival reduction one hour post harvest [44]. The short-term storage of bone grafts can significantly impair their function, highlighting the importance of developing better graft preservation strategies. Previous studies, such as Arabiun et al. [6] demonstrated that grafts stored for extended periods (12 h) exhibited a reduced osteoblast count compared to those stored for shorter periods (2–4 h). These findings emphasize the critical influence of retention time on cellular vitality and the osteogenic activity of the grafts.

Low-temperature preservation is widely used for safeguarding biological tissues by slowing metabolism and extending cellular viability. This method is particularly beneficial for OVX grafts, as OVX cells are more vulnerable to environmental fluctuations. Our study confirmed the advantages of low-temperature preservation across both young and OVX bone grafts, with a more pronounced effect in OVX grafts, where cellular resilience is weaker. In contrast, dry storage conditions are detrimental to cellular metabolism, which has been well documented in the literature [45].

We also investigated the use of platelet-rich plasma (PRP) as a preservation solution. PRP is rich in growth factors that promote angiogenesis, an essential process in early-stage bone regeneration. The use of PRP as a preservation solution was found to be particularly effective, offering osteogenic benefits while being readily accessible and cost-effective. Although blood or PBS can also serve as preservation solutions, they are less effective than PRP. In the absence of PRP, blood or normal saline may be viable alternatives for graft preservation.

While subcutaneous implantation in nude mice provided a controlled and reproducible environment for assessing early osteogenic responses, we acknowledge that this ectopic model lacks key features of the orthotopic bone environment. Specifically, subcutaneous tissues do not replicate the mechanical loading, osseous vascularization, and marrow-derived signaling that occur in native bone [46]. These differences may limit the translational applicability of osteoinductive and osteoconductive outcomes observed in this model. Previous studies have shown that subcutaneous implantation underestimates the mechanical integration and long-term remodeling capacity of bone grafts compared to orthotopic models, such as critical-sized craniofacial or femoral defects. Nevertheless, subcutaneous implantation offers distinct methodological and ethical advantages, particularly in studies requiring extensive histological analysis. For instance, a single nude mouse can receive multiple subcutaneous implants at once, enabling parallel evaluation of several conditions (e.g., different preservation media or temperatures) while minimizing animal use. In contrast, orthotopic implantation typically allows only one graft per animal and often necessitates the sacrifice of a large number of animals to perform multiple stains, including ALP, Runx2, Osteocalcin, and aniline blue. To uphold the 3Rs principle (replacement, reduction, and refinement) in animal research, we strategically employed the subcutaneous model to reduce overall animal usage and refine experimental procedures without compromising data validity. To mitigate its limitations, we further incorporated an orthotopic rat alveolar defect model to more accurately mimic clinical scenarios and validate key findings. This dual-model approach enhances both the translational relevance and ethical rigor of our study design.

While rat models are widely used in bone graft preservation research due to their convenience, cost-effectiveness, and rapid bone healing, their applicability to human clinical practice requires careful interpretation. Significant biological and mechanical differences exist between rat and human bone, including the lack of Haversian systems in rodents, higher metabolic rates, and faster remodeling cycles, which can lead to differences in graft integration and healing outcomes [47,48]. Additionally, rat bones experience lower mechanical loading compared to human long bones, which may influence the remodeling process and the effectiveness of preservation strategies [49]. While findings in rat models provide essential mechanistic insights, their direct translation to human settings remains uncertain, as certain bone graft materials and osteogenic factors, such as recombinant BMP-2, have demonstrated strong efficacy in rodents but inconsistent or diminished results in human trials [47]. Therefore, further validation in larger animal models, such as rabbits, dogs, or minipigs, which possess bone structures more comparable to humans, is necessary before advancing findings to clinical trials (Taguchi et al., 2021; Zhang et al., 2021) [47,50]. Additionally, future studies should aim to incorporate aged or osteoporotic animal models to better mimic human conditions, particularly in populations with compromised bone healing capacity [48]. To improve clinical translatability, comparative studies across species, long-term observation of remodeling patterns, and standardized experimental protocols are crucial. By integrating findings from multiple preclinical models and carefully designing human trials, researchers can bridge the gap between animal studies and effective clinical applications in bone graft preservation [49,50].

There are several limitations to this study. First, animal models, particularly rodents, may not fully replicate human bone biology and graft responses, limiting the generalizability of the findings to human clinical outcomes. Additionally, the study had a short follow-up period of only 21 days, which may not capture the long-term effects of storage conditions on graft survival and integration. Bone remodeling and graft incorporation typically take longer, and the evaluation period may not have been sufficient to assess clinical relevance fully. While the sample size was adequate for preliminary analysis, the lack of statistical power may have affected the ability to detect small differences, particularly when considering age-related variability. Furthermore, while human bone grafts were included in an orthotopic model, the study primarily focused on animal grafts. Human bone grafts are highly variable, influenced by factors such as donor age and health conditions, which were not fully addressed in the study. Future research should incorporate larger, more diverse human bone samples to better assess the clinical applicability of these findings.

## 4. Materials and Methods

The study design encompassed both in vitro and in vivo components, with the objective of investigating the effects of holding time, temperature, and storing solution on the viability and osteogenic potential of bone grafts. The in vitro segment of the study employed rats from two distinct groups, OVX and normal, to explore holding time and age-related impacts on bone graft properties.

The in vivo study was conducted in two parts: implantation of bone grafts into the subcutaneous space of nude mice and orthotopic implantation into alveolar defects in rats. The first part involved ectopic transplantation, in which 1 mm^3^ bone grafts from rats were placed into the subcutaneous space of nude mice. The second part involved the implantation of bone grafts into alveolar defects in rats.

### 4.1. Animal Preparation and Cancellous Bone Graft Harvesting

Cancellous bone grafts were extracted from the femoral bone marrow of Lewis or Wistar rats using a standardized surgical protocol to ensure consistency across samples. To simulate bone regeneration challenges under osteoporotic conditions, ovariectomized (OVX) rats were used as an osteoporotic model. Ovariectomy induces an osteoporotic phenotype. Rats that underwent OVX at 6-week-old showed delayed alveolar bone healing at 14 weeks old, similar to that of 12-month-old rats [51].

All procedures were performed under isoflurane anesthesia (3–5%), and euthanasia was conducted via CO_2_ inhalation followed by cervical dislocation to minimize suffering. After carefully dissecting the hind limbs and exposing the femur while preserving the periosteum, a piezoelectric surgical device (Piezosurgery^®^, Mectron, Carasco, Italy) was used to minimize thermal damage and ensure precise graft cutting. Additionally, a low-speed rotary drill (≤1500 rpm) under continuous saline irrigation was used to prevent overheating and preserve cellular viability. Bone chips were collected using a bone scraper (Medesy, Maniago, Italy), ensuring a standardized 1 mm^3^ graft size for uniform implantation and osteogenic comparisons. To further ensure reproducibility, all grafts were immediately immersed in either saline or PRP at 4 °C, 23 °C, or 37 °C, depending on the assigned experimental condition. Special precautions were taken to minimize air exposure, with grafts transferred within 1 min post-harvest to prevent drying-induced apoptosis. All procedures were conducted under sterile conditions in a biosafety cabinet to prevent contamination.

### 4.2. In Vitro Study

#### 4.2.1. Live/Dead Cell Viability Assays

We employed the Live/Dead Cell Viability Assay Kit (Sigma-Aldrich, St. Louis, MO, USA) to assess the viability of cells seeded on the scaffolds following the manufacturer’s instructions. The Live/Dead staining solution was prepared using 3 μL of 4 mM calcein-AM (excitation 494 nm and emission 517 nm) and 5 μL of 2 mM ethidium homodimer-1 (EthD-1) (excitation 528 nm and emission 617 nm) in 10 mL of PBS. Subsequently, all samples were exposed to 300 μL of the staining solution and incubated for 15 min at 37 °C. Microscopic imaging was then conducted to observe the stained samples.

#### 4.2.2. Alkaline Phosphatase (ALP) Stain

To perform the alkaline phosphatase stain (ALP), the cancellous bone was treated with a fixative solution consisting of citrate working solution and acetone in a 2:3 ratio. Following this, ALP staining was conducted using ALP stain solution (Sigma-Aldrich, St. Louis, MO, USA; 85L2-1KT) and Mayer’s hematoxylin solution. After the staining process, the bone grafts were washed with distilled water and examined under a microscope for documentation.

### 4.3. In Vivo Study

The animal protocol was approved by the Institutional Animal Care and Use Committee (IACUC) of Chang Gung Memorial Hospital, following AAALAC guidelines. A total of 18 rats were used in this study and randomly assigned to three groups (*n* = 6 per group) based on the preservation medium of the bone grafts (PBS, blood, or PRP).

Femurs were harvested from donor rats immediately after euthanasia, with all adherent soft tissue carefully dissected. Bone grafts were meticulously prepared using controlled drilling in a saline solution to minimize thermal damage. Bone chips were extracted using a piezoelectric device operating at a specific frequency and amplitude, with continuous saline irrigation to prevent overheating. Additional bone grafts were extracted using bone-cutting forceps to ensure consistency and reproducibility across samples. Cancellous bone grafts were subsequently handled under different temperature conditions (4 °C, 23 °C, or 37 °C) for 10 min to evaluate their viability and osteogenic potential before implantation or further in vitro analysis.

#### 4.3.1. Subcutaneous Implantation in Nude Mice

All animal experiments adhered to the Guide for the Care and Use of Laboratory Animals of the National Institutes of Health and were approved by the Chang Gung Memorial Hospital review committee. Under general anesthesia using intramuscular ketamine (20 mg/kg), a small dorsal incision was made on nude mice. Prepared bone grafts were implanted into the subcutaneous pocket created through the incision. The incision was then closed primarily and treated with antibiotic ointment to prevent infection. Nude mice were euthanized after 14 or 21 days for graft analysis.

#### 4.3.2. Histological Examinations

The region of interest was dissected and fixed using 10% formaldehyde. Alcohol-based dehydration was performed. The tissue was embedded in paraffin and subsequently sectioned. These sections underwent immunohistochemistry after being subjected to an alcoholic gradient dehydration and rehydration process. For the immunohistochemical analysis, the section was boiled in 10 mM sodium citrate for 20 min, followed by rinsing in 10% H_2_O_2_ for 10 min. Sections were then incubated with primary antibodies against Runx2, Osteocalcin, and Osterix (Abcam, Cambridge, MA, USA) at 4 °C overnight. After rinsing, slides were treated with biotinylated secondary antibodies and the ABC reagent (Vector Laboratories, Newark, CA, USA), followed by chromogenic detection using DAB (3,3′-diaminobenzidine) substrate. Hematoxylin was used for counterstaining. All stained slides were visualized under a bright-field microscope, and images were captured for analysis. Quantification of staining intensity was performed using ImageJ software (National Institutes of Health, Bethesda, MD, USA; version 1.53).

To assess collagen deposition, paraffin-embedded bone sections were stained with aniline blue. Following deparaffinization and rehydration, sections were incubated in Bouin’s fixative at 56 °C for 1 h to enhance staining contrast. After rinsing with running tap water, sections were stained with Weigert’s iron hematoxylin solution for 10 min, followed by Biebrich scarlet-acid fuchsin for cytoplasmic staining. Subsequently, phosphomolybdic-phosphotungstic acid solution was applied for 10 min, followed by aniline blue solution for 5 min to stain collagen. Slides were then differentiated in 1% acetic acid for 2 min, dehydrated, and mounted. Collagen-rich areas stained blue and were quantified by digital image analysis using ImageJ software.

### 4.4. PRP and Blood Preparation

Platelet-rich plasma (PRP) was prepared using freshly isolated whole blood from rats. Blood was collected via cardiac puncture under deep anesthesia and transferred into anticoagulant-coated tubes (citrate or heparin) to prevent clotting.

The first centrifugation (200–300 g, 10–15 min) separated plasma from red blood cells. The plasma fraction was carefully aspirated and subjected to a second centrifugation (1000–1500 g, 10 min) to concentrate the platelets. The resulting PRP layer (buffy coat) was collected and used immediately for graft immersion.

To ensure consistent PRP exposure, bone grafts were immersed in PRP at a standardized ratio of 1 mL per gram of graft. The immersion lasted 5 min at room temperature (23 °C) before implantation. PRP was not stored or frozen prior to use, ensuring the highest platelet viability and growth factor availability.

For control comparisons, grafts were similarly immersed in either fresh blood or phosphate-buffered saline (PBS) under identical conditions.

### 4.5. Orthotopic Implantation in Rat Alveolar Bone Defect

A standardized 1.5 mm diameter defect was created at the edentulous ridge, positioned 1 mm mesial to the first molar (M1) to ensure consistency across specimens. Bone grafts were freshly harvested from donor rats and processed under different handling conditions (4 °C vs. 23 °C, 10 min) before implantation. Each rat received a bone graft into the prepared alveolar defect under general anesthesia. A comprehensive summary of the experimental conditions, methodologies, and key findings from both in vitro and in vivo studies is provided in Supplementary Table S1.

Micro computed tomography (Micro-CT) imaging was nanoScan^®^ SPECT/CT system (Mediso Medical Imaging Systems, Budapest, Hungary) to evaluate calvarial bone regeneration at post-implantation day 1 (PID 1) and day 21 (PID 21). The bone volume fraction (BV/TV, %) was quantified using ITK-SNAP software (version 3.8.0) by analyzing sagittal views of the grafted defects. To ensure consistency, a single operator conducted all calculations to minimize observer bias.

Micro-CT analysis revealed that bone grafts preserved in platelet-rich plasma (PRP) exhibited the highest bone volume fraction at PID 21, followed by those preserved in blood and phosphate-buffered saline (PBS), while grafts left without a preservation solution showed the lowest bone volume. Quantitative data analysis demonstrated the following BV/TV (%) values at PID 21.

The same histological and immunohistochemical staining procedures, including aniline blue, ALP, and IHC staining for osteogenic markers (Runx2, Osteocalcin, and Osterix), were performed on explanted grafts from the in vivo animal models to assess collagen deposition, osteoblastic activity, and osteogenic differentiation.

### 4.6. Statistical Evaluation

The data are expressed as mean ± standard deviation (SD). The sample size was determined based on previous studies assessing bone graft viability, ensuring sufficient statistical power to detect significant differences between experimental groups. A power analysis was conducted using G*Power software (version 3.1.9.7; Heinrich Heine University, Düsseldorf, Germany), with an effect size of 0.8, an *α*-value of 0.05, and a power of 80%, leading to a minimum required sample size of six animals per group.

To analyze differences among multiple groups, the nonparametric Kruskal–Wallis test was chosen, followed by Dunn’s multiple comparison post hoc test. The Kruskal–Wallis test was selected because data distribution did not meet normality assumptions (Shapiro–Wilk test, *p* < 0.05), and the sample sizes were relatively small, making parametric tests like ANOVA less suitable. This approach allows for robust analysis of ordinal and non-normally distributed data, commonly encountered in biological studies involving tissue viability and histological assessments. A *p*-value < 0.05 was considered statistically significant. All statistical analyses were performed using SPSS software 22.0 (SPSS Inc., Chicago, IL, USA) cements.

Although ANOVA is a standard method for comparing group means, due to the relatively small sample size and deviation from normality in our data (as assessed by the Shapiro–Wilk test), we opted for the Kruskal–Wallis test followed by Dunn’s post hoc analysis to increase the robustness and reliability of our statistical interpretations. This non-parametric approach provides greater confidence in the validity of observed differences among groups despite sample size limitations.

## 5. Conclusions

In summary, this study underscores the critical role of minimizing handling time, optimizing storage temperature, and selecting effective preservation solutions in enhancing the viability and osteogenic potential of cancellous bone grafts. Handling times exceeding 10 min significantly compromise graft viability, highlighting the importance of swift intraoperative processing. Storage at 4 °C emerged as the optimal temperature, particularly for OVX grafts, by preserving cellular integrity and enhancing osteogenic activity. Additionally, PRP proved to be a superior preservation solution, outperforming blood and PBS by promoting biomarker expression and bone regeneration, especially in elderly grafts. As a readily available, cost-effective option, PRP holds significant promise for clinical applications. By integrating these strategies, improved bone grafting outcomes can be reliably achieved, advancing patient care and clinical success.

## Data Availability

All authors agree to make the results of this manuscript available for all readers, including links to publicly archived datasets analyzed or generated during the study.

## Supplementary Materials

The following supporting information can be downloaded at: https://www.mdpi.com/article/10.3390/ijms26094255/s1.
