# Supplementary material for: Optimizing the Handling of Cancellous Bone Grafts to Preserve Their Osteogenic Potential in Craniofacial Surgery
Source: Int J Mol Sci. 2025 Apr 30;26(9):4255. doi: 10.3390/ijms26094255 (PMC12072118; doi:10.3390/ijms26094255)
Supplement: Supplementary file 1 [file ijms-26-04255-s001.zip › Supplementary Table S1.pdf]

**Table S1. Summary of Experiments Evaluating Bone Graft Handling Conditions and Outcomes.**

| Experiment Type | Conditions Tested | Methods & Assessments | Key Findings |
|---|---|---|---|
| **In vitro assessment** | Handling time (5, 10, 30 min); Temperature (4°C, 23°C, 37°C) | Live/dead staining, ALP staining | Prolonged handling (>10 min) significantly increased apoptosis. Optimal cell viability and ALP activity observed at shorter times and 4°C temperature. |
| **In vivo - Subcutaneous implantation of bone graft in nude mice** | Preservation medium (PBS, Blood, PRP); Temperature (4°C, 23°C) 10 min | Immunohistochemistry (IHC) at PID 14 (ALP, Runx2, Osterix staining) | PRP preservation at 4°C showed highest osteogenic marker expression and cellular viability. Reduced osteogenic activity at 23°C. |
| **In vivo - Alveolar defect implantation** | Preservation medium (PBS, Blood, PRP); Temperature (4°C, 23°C) 10 min | Micro-CT and IHC (ALP, Runx2, Osteocalcin, Osterix, Aniline blue staining) at PID 21 | PRP-treated grafts exhibited superior bone regeneration, greatest osteogenic potential, and collagen deposition. PBS resulted in significantly lower bone formation. |
